# Holding soft objects increases expectation and disappointment in the Cyberball task

**DOI:** 10.1371/journal.pone.0215772

**Published:** 2019-04-23

**Authors:** Toshiki Ikeda, Yuji Takeda

**Affiliations:** 1 Graduate School of Comprehensive Human Sciences, University of Tsukuba, Tsukuba, Japan; 2 Automotive Human Factors Research Center, National Institute of Advanced Industrial Science and Technology (AIST), Tsukuba, Japan; University of Rome, ITALY

## Abstract

When a person is excluded from a group, she/he often experiences negative emotions, referred to as social pain. Previous studies have reported that touching/holding soft objects can lead to a decrease of negative attitude toward uncertain events, and it is possible that such physical intervention may also be effective for reducing social pain induced by the group exclusion. This study examines the effects of holding soft objects on social pain. Participants held either a soft or hard cushion while performing the Cyberball task, a virtual ball-tossing game that experimentally controls social exclusion. In addition to the subjective rating of social pain, we measured the contingent negative variation (CNV) of event-related potentials, a variable related to perceivers’ expectations about forthcoming events. Results showed that, contrary to our prediction, holding a soft cushion increased the subjective rating of social pain. Furthermore, holding a soft cushion increased the amplitude of CNV while performing the Cyberball task. These results suggest that holding soft objects increases expectations about uncertain forthcoming events, but it does not reduce negative emotion.

## Introduction

Humans are social animals. We need to interact with each other to survive. Indeed, a person who is excluded from a group may often experience negative emotion and is likely in the future to avoid interacting with the other members of the group, resulting in social disadvantages. Exploring the factors affecting negative emotions elicited by social exclusion is important to understand the nature of social pain.

A Cyberball paradigm is a virtual ball-tossing game that experimentally controls social exclusion [1–2]. In this paradigm, a participant throws and catches a ball with two other players on a screen via a computer network. When participants catch the ball, they are required to choose one of two players to whom they will next throw the ball. In a typical Cyberball task, there are two conditions: a *fair-play* condition and an *exclusion* condition. In the fair play condition, the ball will be thrown to an experimental participant and the other two players with equal probability. By contrast, in the exclusion condition, relative to the probabilities of other players ball is thrown with a low probability to the participant. Negative emotions induced by the experiences of exclusion are often referred to as social pain [3].

In previous studies, it has been demonstrated that negative emotion measured by subjective and neurophysiological indices was reduced if emotional support was given [4–8]. For example, Onoda et al. [8] investigated the effect of emotional support by presenting empathetic and concerned text messages (e.g., “Sorry, I know it was unpleasant for you to be excluded”) about the negative emotions induced by social exclusion during the Cyberball task. The results showed that social pain was reduced when emotional support was given.

In addition to emotional support, physical interventions such as touching/holding soft objects might be effective for reducing negative emotions. However, previous studies have not specifically addressed the effects of touching/holding soft objects on social pain. Nevertheless, other studies have reported that touching soft objects such as a soft blanket or a soft fabric decreases negative ratings for other people [9], increases the tolerance for uncertainty [10]. Moreover, holding soft objects such as teddy bears induces an increase in prosocial behavior [11], as well as an increase in task-related motivation [12]. These findings suggest that touching/holding soft objects might reduce negative emotions. The effects of touching/holding soft objects on emotions might be associated with interpersonal touch [10], which plays an essential role in our emotional wellbeing [13].

The effects of touching/holding soft objects on emotional attitudes may be due to the relationship between the metaphor of softness and the state of safety [10]. It is well known that animal infants (including human infants) are likely to seek soft contact from caregivers [14–15]. The lack of contact experiences results in an increase of developmental deficits, impairment of immune function, and adult neurological/psychiatric problems [16]. Given that such association between soft contact and safe experiences in an earlier period of life lasts until adulthood, the effects of touching/holding soft objects on emotional states can be explained by the misattribution of experience of physical safety.

Although there is no direct evidence of that touching/holding soft objects provides emotional support that reduces social pain in the Cyberball task, it remains possible that social pain, induced by exclusion, can be reduced in this manner. On the other hand, because previous studies have demonstrated the increase (decrease) of a positive (negative) attitude in uncertain situations but not after the occurrence of negative events, it is also possible that the safety metaphor of touching/holding soft objects only applies to increases involving expectations about uncertain forthcoming positive events and does not reduce negative emotion after the occurrence of negative events. If so, then touching/holding soft objects would increase rather than decrease social pain induced by exclusion. That is, participants may be more likely to expect that they will be thrown the ball if they touch the soft objects, but when this does not happen, they will be more greatly disappointed. This study examined the effects of touching/holding soft objects in the Cyberball task to clarify this issue.

We measured participants’ subjective ratings of social pain and event-related potentials (ERPs) to examine the effects of touching/holding soft objects on social pain when performing the Cyberball task. We examined the contingent negative variation (CNV) to estimate expectations of being thrown the ball. The CNV is a slow wave occurring in the interval between the presentation of a warning stimulus and an imperative stimulus that requires a motor response [17]. The CNV is known to be related to the subjective probability of stimulus occurrence [18], as well as the expectation and anticipation of the imperative stimulus [19–20]. Therefore, the CNV amplitude reflects the strength of participants’ expectation of being thrown the ball during the Cyberball task.

In addition to the CNV that develops before the onset of the ball movement, we also examined the P3 component that develops after the onset of ball movement. Previous studies have reported that the P3 amplitude is related to the subjective probability of stimulus occurrence, violations of the expectancy of being thrown the ball [21–23], and correlated with the degree of social pain during the Cyberball task [24]. The CNV develops before, and the P3 component develops after the ball movement, and it is reasonable to consider that the CNV reflects preparation and P3 component reflects evaluation processes related to the subjective expectations to be thrown the ball. As a result, it is possible that the CNV amplitude would increase if touching/holding soft objects increases the subjective expectations of being thrown the ball, whereas P3 amplitude would increase if such manipulations would increase the perception of expectancy violation. The CNV is considered to reflect not only the expectation but also the readiness for action [17]. Therefore, we imposed no time pressure to initiate action in this study to minimize the effect of activating readiness for action.

In this study, the participants held either a soft or a hard cushion while performing the Cyberball task, and we instructed them to hold one of the two cushions throughout an experimental block. The Cyberball task comprised two conditions: the fair-play condition (equal probability of the participant and other players receiving the ball), and the exclusion condition (extremely low probability of the participants receiving the ball compared to other players). If holding soft objects decreases negative emotions and the perception of expectancy violation, the subjective rating of social pain and the P3 amplitude were expected to be lower when the participants held the soft cushion than when they held the hard cushion. On the other hand, if holding soft objects only increases preparatory expectations about uncertain forthcoming events without decreasing negative emotions, the CNV amplitude was expected to increase when participants held the soft cushion. Furthermore, it is possible that the subjective rating of social pain of the participants would be affected by holding a soft cushion, such that social pain would increase if the ball is not thrown to them. This is because participants would develop a high expectation of receiving the ball when holding the soft cushion, which would be violated when the anticipated outcome fails to occur.

## Materials and methods

### Participants

Twenty-five healthy participants (3 female, *M*_*age*_ = 22.20, *SD*_*age*_ = 2.74) took part in the experiment. All participants had normal or corrected-to-normal vision.

They received payment (1250 yen / 1 h) at the end of the experiment. The National Institute of Advanced Industrial Science and Technology (AIST) Safety and Ethics committee approved the research protocol. This study was conducted only after each of the participants had given written informed consent.

### Stimuli and procedure

The visual stimuli were presented on a 17-inch cathode ray tube display (Sony, Trinitron Multiscan G220) with the resolution of 1280 × 1024 pixels, which was controlled by Windows 7, MATLAB (MathWorks Inc.), and Psychophysics Toolbox [25–27]. The refresh rate of the display was set to 60 Hz; the viewing distance was approximately 70 cm.

Two visually similar cushions (soft and hard cushions) were used as holding objects. Both cushions were covered with white cotton, and their sizes were 55 × 55 × 10 cm. The soft cushion was made of polyester, and the weight was about 850 g. The hard cushion was made of polyethylene pipes, and the weight was about 1000 g. We instructed participants to hold one of the cushions with their left arm and place it on their thighs.

**Fig 1:**
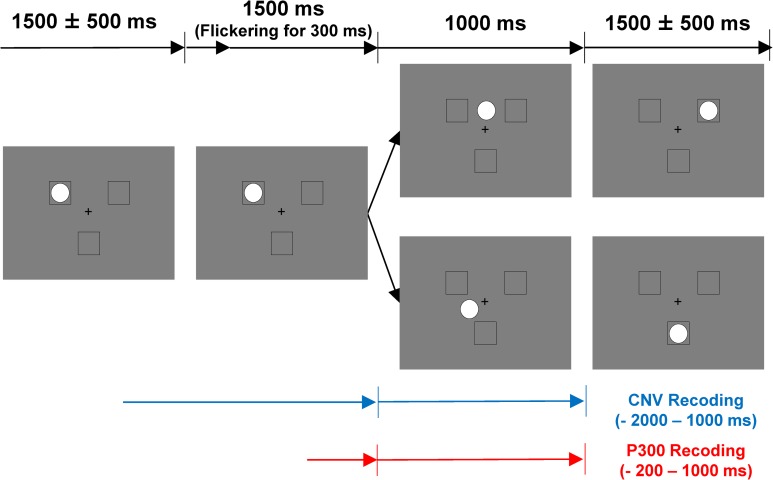
Illustration of the experimental procedure for the cyberball task. Black squares (outlined) indicate players in the Cyberball task. The lower square corresponds to the participant; others correspond to computer players. The ball flickered 1500ms before moving. Then, the ball moved to the participant or to another player. The computer players held the ball for a random period lasting between 1500 ± 500 ms. After the participant received the ball, she/he was required to selectively toss it to the left or right player by pressing the F key or J key. ERPs were computed by time-locking to the ball movement.

Similar to the previous study [19], the Cyberball task was modified to render the timing of the ball tosses clear and discernible suitable for measuring ERPs. Fig 1 illustrates the Cyberball task in this study. Two computer-generated opponents (black outline squares, 2.1° × 2.1° of visual angle) appeared at the top left and the top right corners of the screen (their distance was 6.5° of visual angle). A player controlled by a participant (a black outline square) appeared at the bottom of the screen. The white filled circle represented the ball (diameter with 1.7° of visual angle). To precisely inform participants of the timing of the ball movements, 1500 ms before it moved, the ball flickered for 300 ms (disappearing for 50 ms and appearing for 50 ms, 3 times). The ball visibly traveled (over a distance of 3.25°) for 1000 ms until another player received it. If participants received the ball, they were required to press an F or J key on the keyboard with their right index or middle finger to toss the ball to the left or right computer-generated player, respectively. Previous studies on CNV associated with reaction time tasks have indicated that CNV is related to motor preparation [17]. Therefore, to minimize the effect of motor preparation, no time pressure was imposed on the participants when they tossed the ball to the computer-generated players while holding either a soft or a hard cushion during the task.

At the beginning of experiment we gave participants a cover-story indicating that tossing performance was unimportant because the task was to examine the mental visualization skills. Participants in this study were aware that the other players did not actually exist.

Each participant performed 8 blocks of the Cyberball task. Participants held one type of cushion in the first 4 blocks and they held the other type of cushion in the remaining 4 blocks. The order of soft and hard cushion conditions was counterbalanced among participants. Participants started the experiment after practicing ten trials of the Cyberball task without holding any cushion.

Each cushion condition consisted of three fair play blocks and one exclusion block. In the fair play block, a participant received the ball 20 times (inclusion) and then tossed it to one of the computer-generated opponents 20 times. Also, in this condition, the computer-generated opponents tossed the ball to each other 20 times (micro-rejection). Thus, each fair play block consisted of 60 trials. In the exclusion block, the participant received and tossed the ball only once, and the computer-generated opponents tossed the ball to each other 60 times (exclusion). Thus, each exclusion block consisted of 62 trials. At the beginning of each cushion condition, participants performed the fair play block. In the remaining three blocks, they performed one exclusion block and two fair play blocks in a random order.

Similar to a previous study [24], during short breaks between the blocks, participants were required to evaluate their mental state by the following questions: “I felt liked,” “I felt rejected,” “I felt invisible,” and “I felt powerful.” These questions were rated on a scale ranging from 1 to 9 (Not At All to Very Much). Two questions, “I felt liked” and “I felt powerful,” were reverse scored such that higher scores for each question indicated a greater level of social pain. We averaged these four questions as the subjective rating of social pain scores. A debriefing was done after the end of the eighth block. The total experiment time was about 50 minutes.

### EEG recording and analysis

The EEG signals were acquired with a digital amplifier (Brain Products, BrainAmp standard system). The silver-silver chloride electrodes were placed at 27 scalp sites: Fp1, Fp2, F7, F3, Fz, F4, F8, FCz, T3, C3, Cz, C4, T4, CPz, T5, P3, Pz, P4, T6, PO7, PO3, POz, PO4, PO8, O1, Oz, and O2, according to the extended international 10–20 system, with AFz as the ground electrode. The EEGs were re-referenced to mathematically averaged earlobes (A1—A2) offline. To monitor blinks and eye movements, vertical and horizontal electrooculograms (EOGs) were acquired using electrodes placed above and below the right eye, and the outer left and right canthi, respectively. The impedance of all electrodes was kept below 10 kΩ. The EEGs and EOGs were digitized at a sampling rate of 1000 Hz and the time constant was set at 10 s. All EEG and EOG signals were low-pass-filtered at 30 Hz with a second-order Butterworth filter to compute the CNV, whereas the 0.1–30 Hz bandpass filter was adopted to compute the P3 component.

The time epochs were set at -2000 ms to 1000 ms and -200 ms to 1000 ms relative to the onset of the ball movement in order to compute the CNV and the P3 respectively. To compute ERPs, we used the epochs in which computer-generated players threw the ball (i.e., the epochs in which participants threw the ball were excluded from the ERP analyses). The independent component analysis was adopted to remove eye-blink-related components, using EEGLAB version 14.1.1b [28]. The epochs in which the signal changes exceeded ± 80 μV on any of the EEGs were excluded from the analysis (13% of epochs on average in CNV; 5% of epochs on average in P3). For the CNV, on average, 104.2 epochs and 52.5 epochs were used for computing the waveforms in the fair play and exclusion conditions respectively. The amplitudes of CNV were evaluated relative to the baseline (the mean amplitude of -2000 to -1500 ms window). For the P3, on average, 56.9 epochs were used for computing each waveform. The amplitudes of P3 were evaluated relative to the baseline (the mean amplitude of -100 to 0 ms window).

The CNV was estimated by the mean amplitude between -1000 and 0 ms at FCz. The P3 was estimated by the mean amplitude between 250 and 450 ms at the central-parietal sites (the averaged waveform of Cz, CPz and Pz). These time windows were determined from visual inspection of the grand mean waveforms in this study.

Mean amplitudes in these time windows were exported and analyzed using SPSS (version 25, IBM). A two-way repeated measure analysis of variance (ANOVA) where conditions of Cushion (soft vs. hard) and Ball (fair play vs. exclusion) were assessed using the mean amplitude of the CNV and the subjective rating of social pain. Also, a two-way repeated measure ANOVA with Cushion (soft vs. hard) and Ball (inclusion vs. micro-rejection vs. exclusion) was used to assess the mean amplitude of the P3. Degrees of freedom were corrected according to Greenhouse-Geisser. Post-hoc analyses with Bonferroni correction were performed when appropriate. The significance level was set at 5%. Note that, we did not distinguish between inclusion and micro-rejection in the analysis of CNV because the temporal region of interest was the time window before the onset of ball movement.

## Results

Due to a high number of artifacts in the EEG, one male participant (21 years old) was excluded from the analyses. Thus, we used the data from 24 participants.

### Subjective ratings

**Fig 2:**
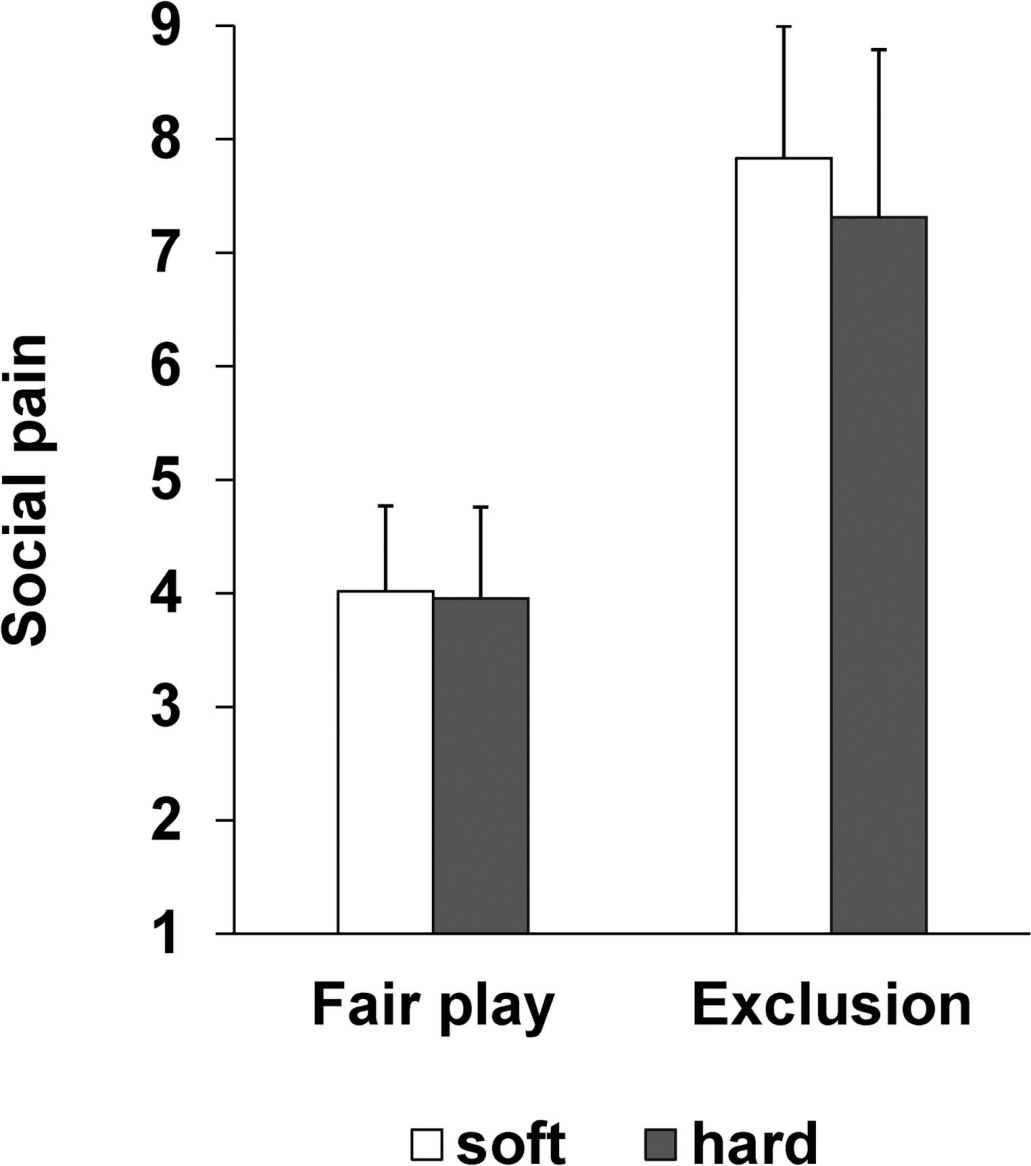
The subjective rating score of social pain. A higher score indicates greater social pain. Error bars indicate the standard deviation across participants.

The subjective rating scores of social pain in each condition are depicted in Fig 2. A Cushion (2) × Ball (2) ANOVA revealed a significant main effect of Cushion (*F* (1, 23) = 6.47, *p* < .05, η_p_^2^ = .22), and a significant main effect of Ball (*F* (1, 23) = 139.05, *p* < .001, η_p_^2^ = .86). The score was higher in the soft cushion condition than in the hard cushion condition (*M* = 5.93 vs. *M* = 5.63), and in the exclusion condition than in the fair play condition (*M* = 7.57 vs. *M* = 3.99). There was no significant interaction (*F* (1, 23) = 2.41, *p* = .13, η_p_^2^ = .10).

### ERPs

**Fig 3:**
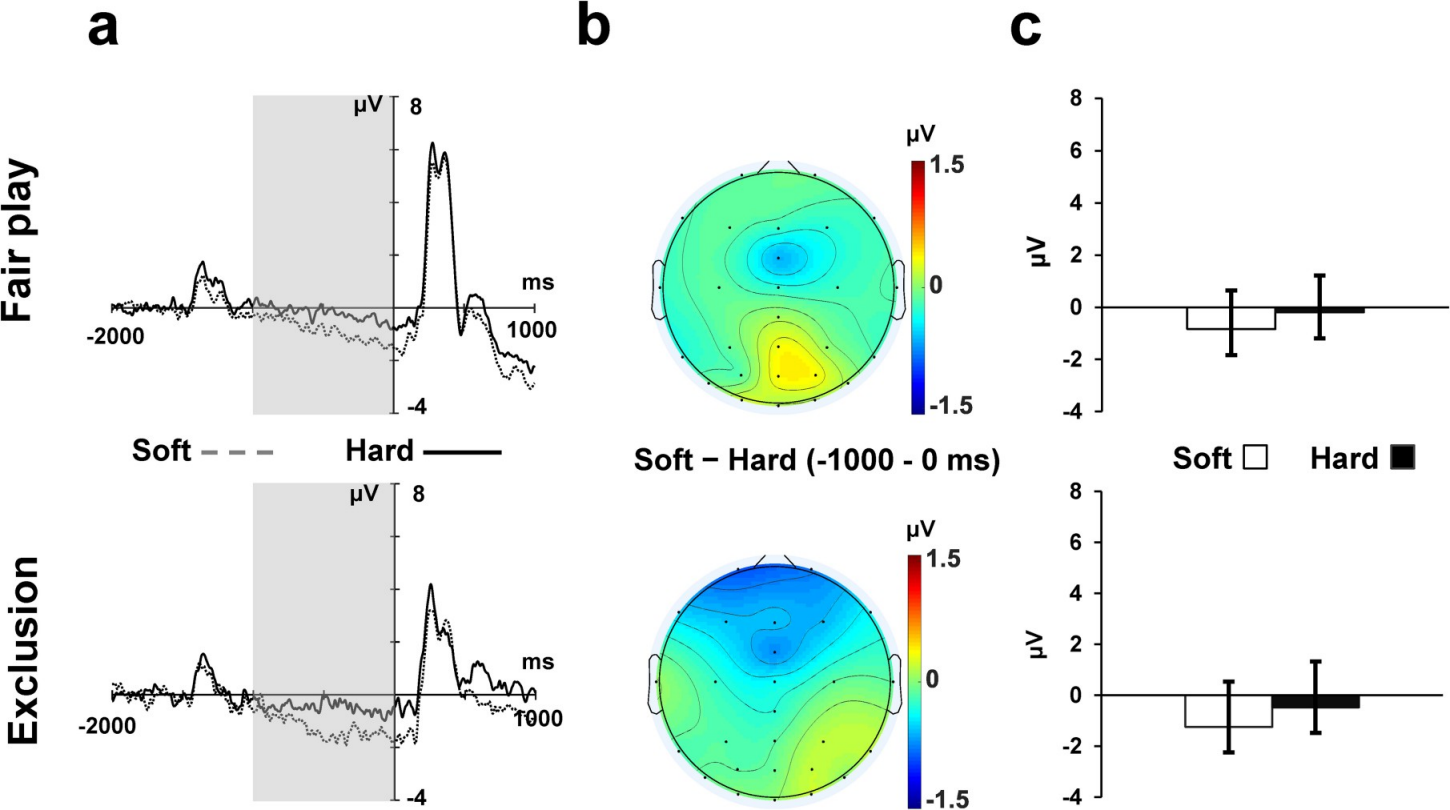
CNV data. (A) Grand-averaged CNV at FCz for Cushion × Ball. (B) Topographical maps representing mean amplitudes of the CNV range (-1000 ms—0 ms). (C) The mean amplitude in each condition. Error bars indicate the standard deviation across participants.

The grand-averaged CNV waveforms that appeared before the ball movement in each condition are depicted in Fig 3. A Cushion (2) × Ball (2) ANOVA revealed a significant main effect of Cushion (*F* (1, 23) = 9.56, *p* < .001, η_p_^2^ = .29). The CNV was more negative in the soft cushion condition than in the hard cushion condition (*M* = -1.04 μV vs. *M* = -.34 μV). There was neither a main effect of Ball (*F* (1. 23) = 1.06, *p* = .31, η_p_^2^ = .04) nor a significant interaction (*F* (1. 23) = .06, *p* = .81, η_p_^2^ = .01).

**Fig 4:**
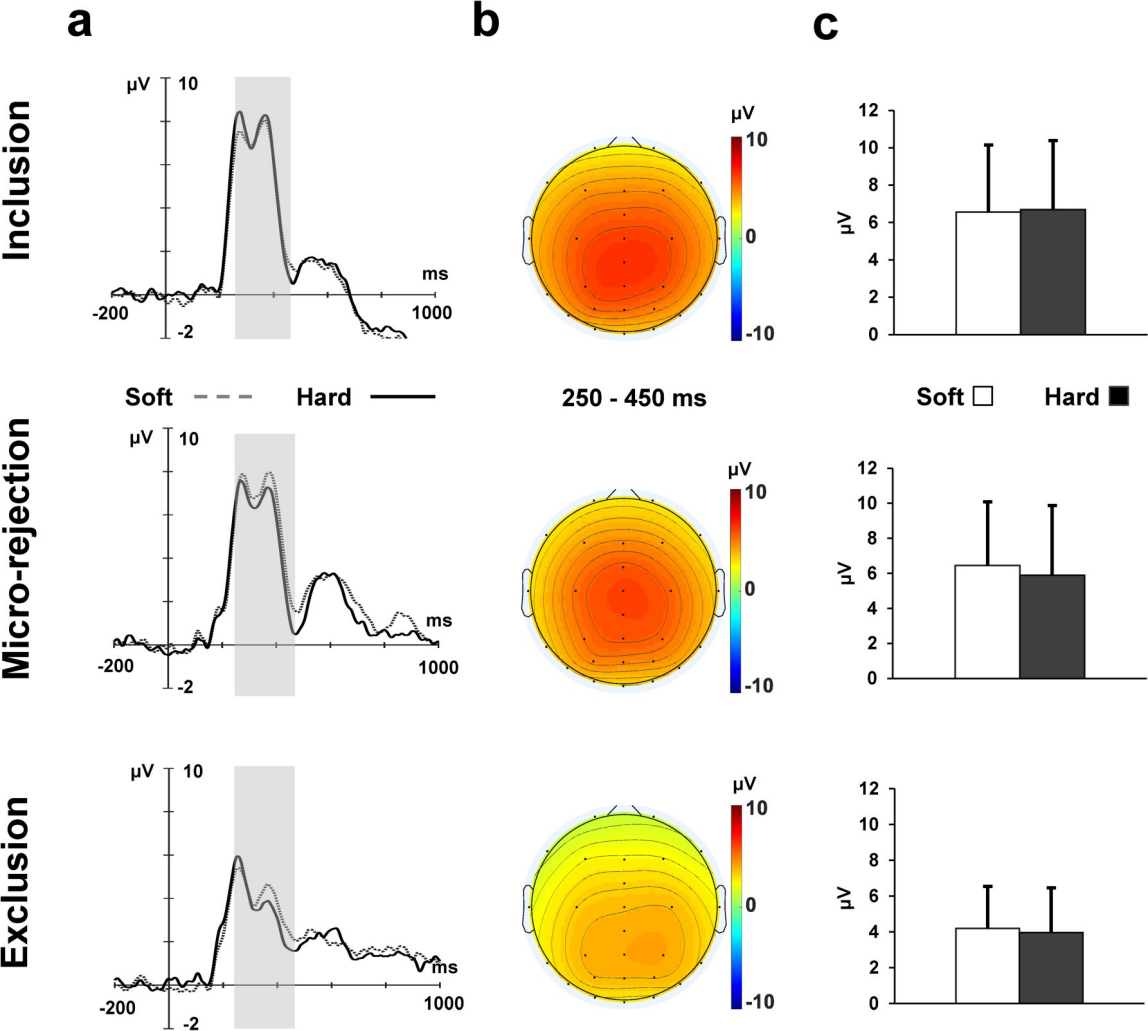
P3 data. (A) Grand-averaged P3 at central and parietal sites (i.e., averaged across Cz, CPz and Pz) for Cushion × Ball. (B) Topographical maps representing mean amplitudes (collapsed across the soft and hard cushion conditions) of the P3 range (250 ms—450 ms). (C) The mean amplitudes of each condition. Error bars indicate the standard deviation across participants.

The grand-averaged P3 waveforms elicited by the ball movement in each condition are depicted in Fig 4. A Cushion (2) × Ball (3) ANOVA revealed a significant main effect of Ball (*F* (1.48, 34.01) = 14.90, *p* < .001, η_p_^2^ = .39). Post-hoc analyses, using Bonferroni correction, indicated significantly larger amplitudes in the inclusion condition than in the exclusion condition (*M* = 6.62 μV vs. *M* = 4.08 μV), and significantly larger amplitudes in the inclusion condition than in the micro-rejection condition (*M* = 6.17 μV vs. *M* = 4.08 μV). There was neither main effect of Cushion (*F* (1. 23) = .19, *p* = .67, η_p_^2^ = .01) nor significant Cushion × Ball interaction (*F* (2. 46) = .72, *p* = .49, η_p_^2^ = .03).

## Discussion

The purpose of the current study was to investigate the effects of holding soft objects on participants’ emotion during the Cyberball task. To this end, we measured variations of the participants’ subjective ratings of social pain and also the amplitudes of CNV and P3 components, while the participants held soft/hard cushions. The results showed that the subjective rating scores of social pain and the amplitudes of CNV were greater when participants held the soft cushion than when they held the hard cushion, irrespective of the fair-play/exclusion conditions. Interestingly, no effect of the cushion was found in P3.

The amplitude of the CNV is related to the preparatory expectation and the anticipation of an imperative stimulus [20]. Therefore, we suggest that the increases of the CNV amplitude in the soft cushion condition reflected the higher preparatory expectation of a participant that the computer-generated players would throw the ball to him/her. Similarly, the increase of subjective social pain in the soft cushion condition can be attributed to the disappointment about exclusion, because participants had developed increased preparatory expectations that they would receive the ball. The results suggest that the CNV component mainly reflected the subjective probability in each conditional context, but not the actual probability of being thrown the ball during the Cyberball task, because the CNV amplitude was not affected by the Ball condition.

The P3 amplitude was larger in the inclusion and micro-rejection conditions than in the exclusion condition. This result is consistent with the findings of a previous study in which a larger P3 was observed in the fair-play condition than in the exclusion condition [24]. However, there was no effect of holding the soft cushion on P3. Previous studies have suggested that the P3 amplitude during the Cyberball task was mainly related to the expectancy violation of being thrown the ball, or the post-stimulus evaluation process. [23]. The amplitude of P3 was not affected by the Cushion conditions, which suggested that post-stimulus evaluative activity represented by the P3 might not be affected by holding soft objects.

The touching/holding soft objects have been considered to act metaphorically as a source of safety and security, hence resulting in a decrease of negative attitude toward uncertain events and other people [9–10]. Furthermore, previous studies have reported that haptic sensations such as body contacts or substitutes for body contact could reinforce this sense of safety and security, which then putatively encourages approaching behaviors [14–15]. In contrast to these studies, the present results showed that holding soft objects did not increase positive emotions; instead, it increased negative emotions. As mentioned previously, holding soft objects may increase preparatory expectations about uncertain events but not necessarily evoke positive emotions. The CNV results of this study supported the latter notion. In this case, when negative feedback was given, holding soft objects appear, ironically, to induce the opposite effect.

Certain limitations of this study should be mentioned here. Firstly, it is unclear why the P3 amplitude was not affected by holding soft objects. As mentioned in the Introduction, because the P3 component develops after the ball movement, it might reflect the evaluation processes that are related to subjective expectations, whereas the CNV might reflect the preparatory processes that are related to subjective expectations. The present results indicated that holding soft objects could affect preparatory but not evaluatory processes. It is suggested that the association between holding soft objects and subjective expectation should be examined in more detail to further clarify the effects of holding soft objects on social pain. Secondly, this study indicated that holding soft objects increased both the subjective ratings of social pain and the CNV amplitude, but it did not demonstrate a direct relationship between social pain and the CNV amplitude. Unfortunately, we could not analyze the correlation between social pain and CNV amplitude, because the number of participants was too small to perform correlation analyses as a between-participants factor. Therefore, the causal relationship between social pain and CNV amplitude remains unclear. Finally, although we used soft and hard cushions of the same size and texture, their weight and heat radiation were slightly different. Therefore, we cannot rule out the possibility that the weight and/or the warmth, rather than the softness affected subjective expectancy during the Cyberball task. Indeed, previous studies have reported that physical weight was related to positive ratings for other people [9] and physical warmth was related to a decrease in the subjective ratings of social pain during the Cyberball task [29]. Further studies examining the effects of weight and warmth are required to address these issues.

In summary, we investigated the effects of holding soft objects on emotion during the Cyberball task. The results suggested that holding soft objects increase the expectations about uncertain forthcoming events, increasing the subjective ratings of social pain if negative feedback is given.

## Supporting information

S1 Table Raw data. (XLSX)
